# Comparing two autologous bone grafting techniques to treat clavicular midshaft atrophic nonunion: a retrospective study

**DOI:** 10.1186/s10195-025-00828-z

**Published:** 2025-02-28

**Authors:** Teng Ma, Qiang Huang, Chaofeng Wang, Cheng Ren, Yibo Xu, Hua Lin, Kun Zhang, Congming Zhang, Zhao Li

**Affiliations:** Department of Severe and Poly Trauma, Honghui Hospital, Xi’an Jiaotong University, 555 Youyi Road, Xi’an, 710054 China

**Keywords:** Autologous bone grafting, Atrophic nonunion, Revision, Superior plate

## Abstract

**Background:**

Open reduction, superior plate (SP) fixation, and autologous cancellous granular bone grafting (ACGBG) are common strategies for treating clavicular midshaft atrophic nonunion (CMAN). We aimed to compare the radiological findings and clinical effects of two autologous cancellous bone grafts (ACBGs) and those of single SP fixation, to treat CMAN.

**Methods:**

This retrospective study comprised 62 patients admitted to our hospital with CMAN (ACGBG with single SP fixation between March 2012 and October 2017, 32 patients; autologous cancellous structured bone grafting [ACSBG] with single SP fixation between November 2017 and May 2021, 30 patients). Patient visual analog scale (VAS) scores for pain and disability of the arm, shoulder, and hand (DASH) scores, obtained preoperatively and at final follow-up, were recorded and analyzed. Statistical differences between the ACGBG and ACSBG groups were assessed using Fisher’s exact and two-sample independent *t* tests.

**Results:**

No statistically significant differences were observed between the two groups in terms of patient demographics or the incidence of complications. VAS and DASH scores decreased significantly from the preoperative day to 9 months postoperatively in both groups, but this difference was not statistically significant at final follow-up. However, at 3 and 6 months postoperatively, compared with mean VAS and DASH scores in the ACGBG group, the ACSBG group showed lower pain and dysfunction scores (*p* < 0.05). The mean fracture healing times were 15.2 (range, 12–20) and 18.6 (range, 12–32) weeks in the ACSBG and ACGBG groups, respectively (*p* = 0.01). One case of plate breakage occurred in the ACGBG group at 5 months postoperatively, with recovery following ACSBG revision with single SP fixation.

**Conclusions:**

ACSBG combined with single SP fixation is a promising and effective alternative technique for promoting bone union and postoperative early functional rehabilitation in treating CMAN.

**Level of evidence:**

Level 3.

## Introduction

The clavicle is a commonly fractured bone, accounting for 2.6–4% of all adult fractures [1]. Fractures of the midshaft clavicle represent 69–82% of all clavicle fractures [1, 2]. Conventionally, the use of a broad-arm sling or figure-of-eight bandaging has been the most frequently used method to treat clavicle fractures [3]. However, the incidence of clavicular nonunion after nonoperative treatment has been reported to be 0.1–5% [4]. In addition, the incidence of nonunion in these fractures increases by up to 2.6–8% after operative treatment [5, 6]. Clavicular nonunion can lead to persistent pain and loss of shoulder function [7].

Compared with acute clavicle fractures, the surgical treatment of clavicle nonunion has a relatively high complication rate and unsatisfactory clinical results, which is a challenge for surgeons [8–10]. Open reduction, superior plate (SP) fixation, and bone grafting allow early recovery and provide good outcomes, and are the most accepted methods for treating clavicle nonunion in patients with atrophic fractures [11]. Autogenous iliac crest bone grafts are considered the gold standard, owing to their osteogenic, osteoconductive, and osteoinductive properties [12]. Cancellous bone is packed into the nonunion gap, which is the most common technique for clavicle nonunion to promote fracture union; however, the incidence of nonunion in long-term follow-up studies has been reported to be 3.1–10.5% [9, 13, 14]. Mechanical instability between the two fractured ends may explain this type of revision failure. Lately, some surgeons have used an additional structured autologous cortical graft as a strut placed under the nonunion site to bridge over the gap and provide mechanical stability for aseptic nonunion of the clavicle [15, 16]. Two studies have reported that, in all but one case of nonunion, solid bone union was achieved at a mean of 14–16 weeks [15, 16]. These studies suggest that enhancing the mechanical stability of both fracture ends is a promising method for treating clavicular nonunion.

In recent years, open reduction and single superior plate (SP) fixation and structured bone grafting has been considered to be a standard technique for treating atrophic nonunion of the clavicle in our hospital, which has achieved good clinical outcomes [17]. In this study, we aimed to compare the radiological and clinical results of treating primary clavicular midshaft atrophic nonunion (CMAN) between the autologous cancellous granular bone grafting (ACGBG) and autologous cancellous structured bone grafting (ACSBG) techniques to provide surgeons with an optimal alternative when treating patients with this type of nonunion.

## Methods

### Patients

Inclusion criteria comprised patients: (i) aged 18–65 years; (ii) who underwent surgery for clavicular midshaft fracture repair at least 9 months prior, for whom the fracture had shown failure to heal for 3 months [18]; (iii) with pain or instability over the local site requiring surgical intervention; and (iv) with sclerosis of the fracture end and no callus formation. Exclusion criteria comprised patients: (i) aged < 18 years or > 65 years; (ii) treated using the ACGBG technique with an intramedullary nail or external fixation; (iii) with local chronic infection of nonunion or hypertrophic nonunion; (iv) with a follow-up time of < 1 year; and (v) with a history of steroid use, metabolic disorders, immunological diseases, or severe cardiovascular diseases.

This was a retrospective study of the medical records and radiographs of all consecutive patients who had undergone surgical intervention for CMAN between March 2012 and May 2021 at the Department of Orthopedic Trauma (Table 1). We divided 62 patients into two groups, namely, 32 patients who had been treated with ACGBG and single SP fixation (ACGBG group) and 30 patients who had been treated with ACSBG and single SP fixation (ACSBG group). All autologous cancellous bone grafts (ACBGs) were harvested from the ipsilateral anterior iliac crests.

**Table 1:** Demographics of patients with CMAN treated by ACGBG and ACSBG

Group	*n*	Age ($\overline{{\text{X}}}$ ± SD, years)	Gender		Affected side		Mode of injury		Complications	
			Male	Female	Left	Right	Fall	Traffic accidents	Yes	No
ACGBG	30	42 ± 12.7	18	12	20	10	21	9	4	26
ACSBG	29	43 ± 14.4	13	16	18	11	19	10	3	26
*T*-value		−0.156								
*P*-value		0.877	0.301		0.789		0.785		1.000	

Demographic and clinical data were collected and evaluated preoperatively. Infection indicators included complete blood count results, C-reactive protein concentrations, and erythrocyte sedimentation rates, which were evaluated to exclude potential infections at the local bone nonunion site. Surgery-related complications were also evaluated.

This study was approved by the ethics committee (202,112,003) at our institution. All patients agreed to publication of their clinical data and signed an informed consent form.

### Operative techniques

**Fig. 1:**
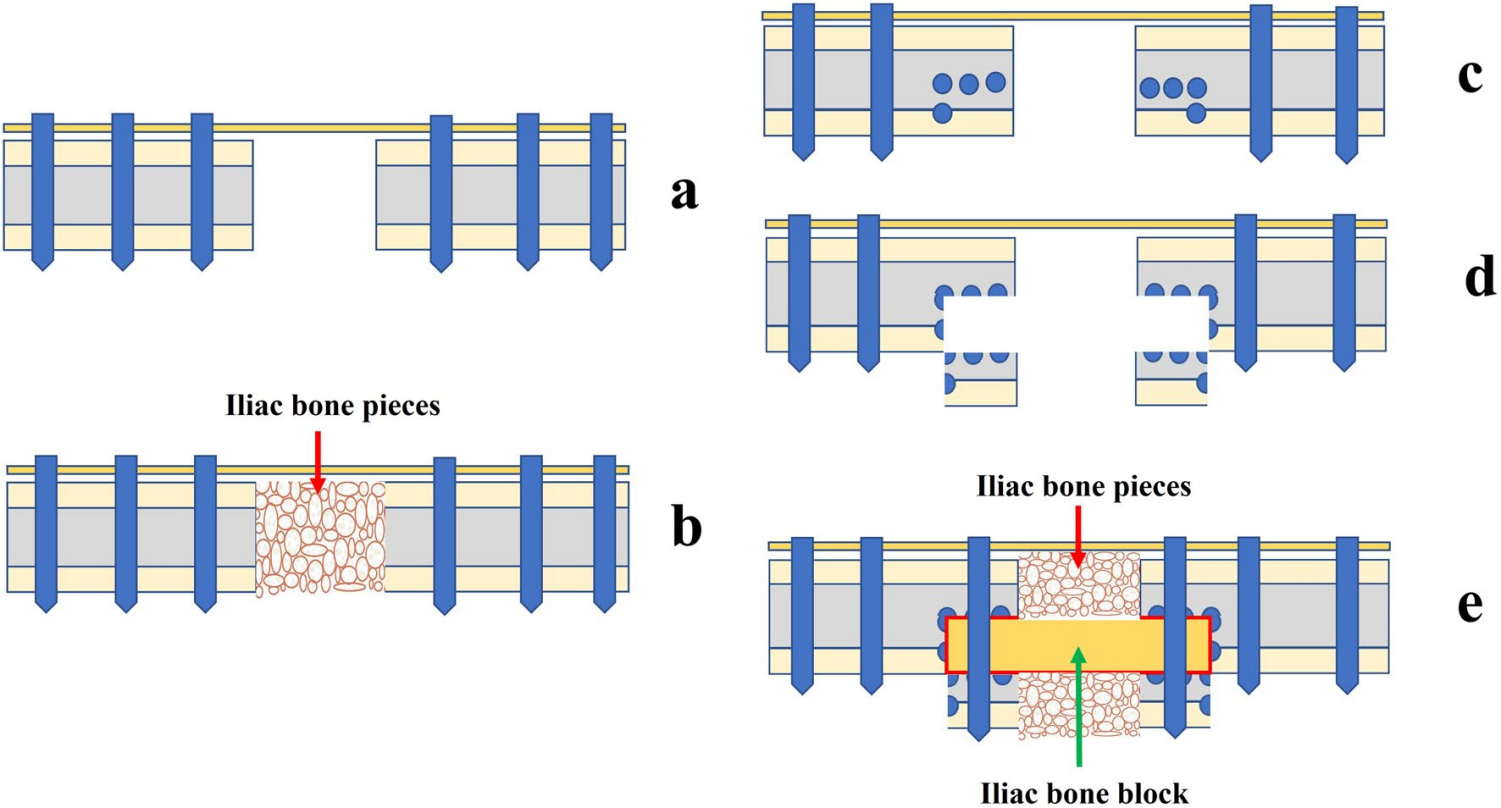
Schematic images of the surgical techniques used in the ACGBG and ASGBG groups. **a** The plate-bone construct prior to bone grafting in the ASGBG group. **b** The final construct of osteosynthesis with the ACGBG technique. **c** The position of drilling bicortical holes (marked with a blue circle). **d** Making an artificial bone groove through moving the fracture block down. **e** The final construct of osteosynthesis using the ACSBG technique. ACGBG, autologous cancellous granular bone grafting; ACSBG, autologous cancellous structured bone grafting

After general anesthesia, the patients were positioned on a beach chair. Each surgical incision was made according to the previous surgical approach to decrease local trauma. The soft tissue was protected through careful dissection and the original internal plate was removed. Scar tissue and sclerotic bone between the medial and lateral ends of the clavicular fragments were thoroughly removed. Bone-holding forceps were used to complete the preliminary reduction by correcting the rotation, angular deformity, and length. The remaining operational steps from the two groups differed, as follows:

In the ACGBG group, a 3.5 mm anatomical reconstruction plate was selected to fix both fracture ends superiorly, leaving at least three screws on each side of the fragments (Fig. 1a). The lengths of the bone defects were measured and recorded. The medullary cavity of both ends was opened with a 2.5 mm drill, then the bone defect was filled using ACGBG.

In the ACSBG group, two screws were inserted into both ends of the plate to maintain alignment and the gap at both fracture segments, followed by measurement of bone defect length. A 2.0 mm Kirschner wire was then used to create a row of bicortical holes along the long axis of both bone nonunion segments until blood seepage (paprika sign) was observed in the holes [19] (Fig. 1c). The row of holes was then extended a further 0.5 cm at both ends, as previously described [17]. A straight osteotome was used to osteotomize the bone block through the lateral cortex along the drill holes and an artificial bone groove was created by moving the fracture block down (Fig. 1d). Subsequently, the ACSBG technique was performed. The detailed process of bone grafting is described below, based on the different operational steps.

A longitudinal incision was made along the iliac crest, the length of which was based on assessment of the bone defect. A bisecting line of the superior cortex of the iliac crest was created using an osteotome, and a corresponding bicortical ilium, sized 4–6 cm, was harvested from the donor site. The periosteum and soft tissue of the iliac bone block were completely removed, and a bone graft was implanted into the bone groove. The method of bone grafting also differed between the two groups. In the ACGBG group, the cancellous and cortical bones were trimmed into granular pieces and packed into the nonunion gap (Fig. 1b). In the ACSBG group, a complete iliac bone block was implanted into the bone groove, according to the length of the bone defect. The autologous iliac bone block and clavicular cortex were fixed to a steel plate using a screw. The remaining bone defects within the gap were filled with autologous cancellous bone pieces (Fig. 1e).

All procedures were performed by one experienced orthopedic surgeon.

### Postoperative treatment

After surgery, antibiotics were infused intravenously within 24 h to prevent wound infection. The affected limb was fixed using a neck–wrist sling for 2–3 weeks. During suspension, each patient’s affected arm was allowed to perform a gentle pendulum movement with the help of the healthy hand. After 3 weeks, each patient started passive shoulder abduction. Activation of the shoulder joint was initiated at 6 weeks postoperatively. Patients were not permitted to lift heavy weights until continuous sclerotic callus formation between the two fracture sites was radiographically confirmed.

### Clinical and radiological assessment

Follow-up visits were scheduled at 4, 8, 12, 16, and 20 weeks, and at 6, 12, 18, 24, and 30 months postoperatively. Pain was assessed using visual analog scale (VAS) scores, and functional outcomes were evaluated using disabilities of the arm, shoulder, and hand (DASH) scores. The VAS and DASH scores of all patients in the ACGBG group collected prior to surgery, and at 3, 6, and 9 months postoperatively, were compared with those in the ACSBG group. Complications associated with each surgical method, including wound infection, skin issues, donor site pain, and venous thrombosis, were recorded and compared at the last follow-up visit. Radiographic evaluation in a standard anteroposterior (AP) view was performed on postoperative day 2 and monthly thereafter until fracture union was confirmed. Union was defined as a completely bridging bone callus passing through the fracture gap in the standard clavicular AP view.

### Statistical analysis

All statistical analyses were performed using SPSS (version 19.0; SPSS Inc., Chicago, IL, USA) software. The results are presented as mean and standard deviation. All data were tested for normality using a Shapiro–Wilk statistical test. Fisher’s exact test was used to analyze demographics, including sex, affected side, mode of injury, and the incidence of complications. A two-sample independent *t*-test was used to assess differences in age and in pre- and postoperative pain VAS and DASH scores. The significance level was set at *P* = 0.05.

## Results

### Patient demographic data

Between March 2012 and May 2021, 62 patients had undergone CMAN surgery. In the ACGBG group, 2 patients were lost to follow-up (total, *n* = 30 patients; 18 men, 12 women; minimum follow-up for retrospective review, 18 months). In the ACSGB group, 1 patient was lost to follow-up (total, *n* = 29 patients; 13 men, 16 women; minimum follow-up for retrospective review, 15 months). The mean ages in the ACGBG and ACSBG groups was 42 ± 12.7 and 43 ± 14.4 years, respectively. In the ACGBG group, there were 20 and 10 left- and right-sided bone nonunion lesions, respectively. In the ACSBG group, there were 18 and 11 left- and right-sided bone nonunion lesions, respectively. In the ACGBG group, a history of falls was the most common mechanism of trauma affecting of 21/30 (70%) patients, followed by traffic accidents. In the ACSBG group, falls accounted for 65.5% (19/29) of injuries, followed by traffic accidents. All metalwork failures resulted from metal fatigue owing to bone nonunion. A comparison of the above results in both groups showed no significant differences. The results are summarized in Table 1.

### Clinical and radiological results

The two groups were similar in terms of average blood loss, operative times, and follow-up times. None of the patients in either group required a blood transfusion. The average bone defect lengths were 2.4 ± 0.6 and 2.6 ± 0.6 cm in the ACGBG and ACSBG groups, respectively (*P* = 0.285), and the difference was not statistically significant (Table 2).

**Table 2:** Clinical and radiological outcomes of patients with CMAN treated by ACGBG and ACSBG

Group	*n*	Operative time (minutes)	Length of bone defect (cm)	Time of bone union (weeks)
ACGBG	30	132.3 ± 20.2	2.4 ± 0.6	18.6 ± 5.4
ACSBG	29	140.2 ± 20.89	2.6 ± 0.6	15.2 ± 3.0
*T*-value		−1.479	−1.078	
*Z*-value				−2.575
*P*-value		0.145	0.285	0.010

In the ACGBG group, both VAS and DASH scores showed clear improvement: the mean VAS score improved from 4.8 ± 1.8 preoperatively to 1.8 ± 1.1 at 9 months postoperatively; the mean DASH score improved from 27.3 ± 6.3 preoperatively to 7.5 ± 4.1 at 9 months postoperatively. Similarly, the mean VAS score improved from 4.7 ± 1.6 preoperatively to 1.7 ± 1.0 at 9 months postoperatively. In the ACSBG group, at 9 months postoperatively, the mean DASH score improved from 26.6 ± 7.4 preoperatively to 7.9 ± 4.9. In the ACGBG group, at 9 months postoperatively, the mean pain VAS and DASH scores were similar to those in the ACSBG group (*P* > 0.05; Table 3). However, some differences were observed between the mean pain VAS and DASH scores at 3 and 6 months postoperatively. In the ACSBG group, at 3 months postoperatively, the mean VAS score decreased by 29% when compared with the ACGBG group (*P* = 0.023, Table 3). However, the mean DASH score in the ACGBG group was higher than that in the ACSBG group (*P* = 0.011; Table 3). Similarly, at 6 months postoperatively, when compared with the ACGBG group, the mean VAS score in the ACSBG group was significantly reduced (*P* = 0.017; Table 3). Moreover, the mean DASH score in the ACGBG group was also lower than that in the ACSBG group (*P* = 0.021; Table 3).

**Table 3:** VAS and DASH scores of patients with CMAN treated by ACGBG and ACSBG

Group	*n*	VAS score				DASH score			
		Pre-operatively	Postoperatively (months)			Pre-operatively	Postoperatively (months)		
			3–	6–	9–		3–	6–	9–
ACGBG	30	4.8 ± 1.8	3.1 ± 1.1	2.8 ± 1.3	1.8 ± 1.1	27.3 ± 6.3	12.2 ± 3.3	10.9 ± 3.1	7.5 ± 4.1
ACSBG	29	4.7 ± 1.6	2.4 ± 0.9	2.0 ± 1.0	1.7 ± 1.0	26.6 ± 7.4	9.8 ± 4.0	8.8 ± 3.6	7.9 ± 4.9
*T*-value		−0.47	2.020	2.453	0.315	0.547	2.615	2.368	−0.092
*P*-value		0.962	0.023	0.017	0.754	0.586	0.011	0.021	0.927

**Fig. 2:**
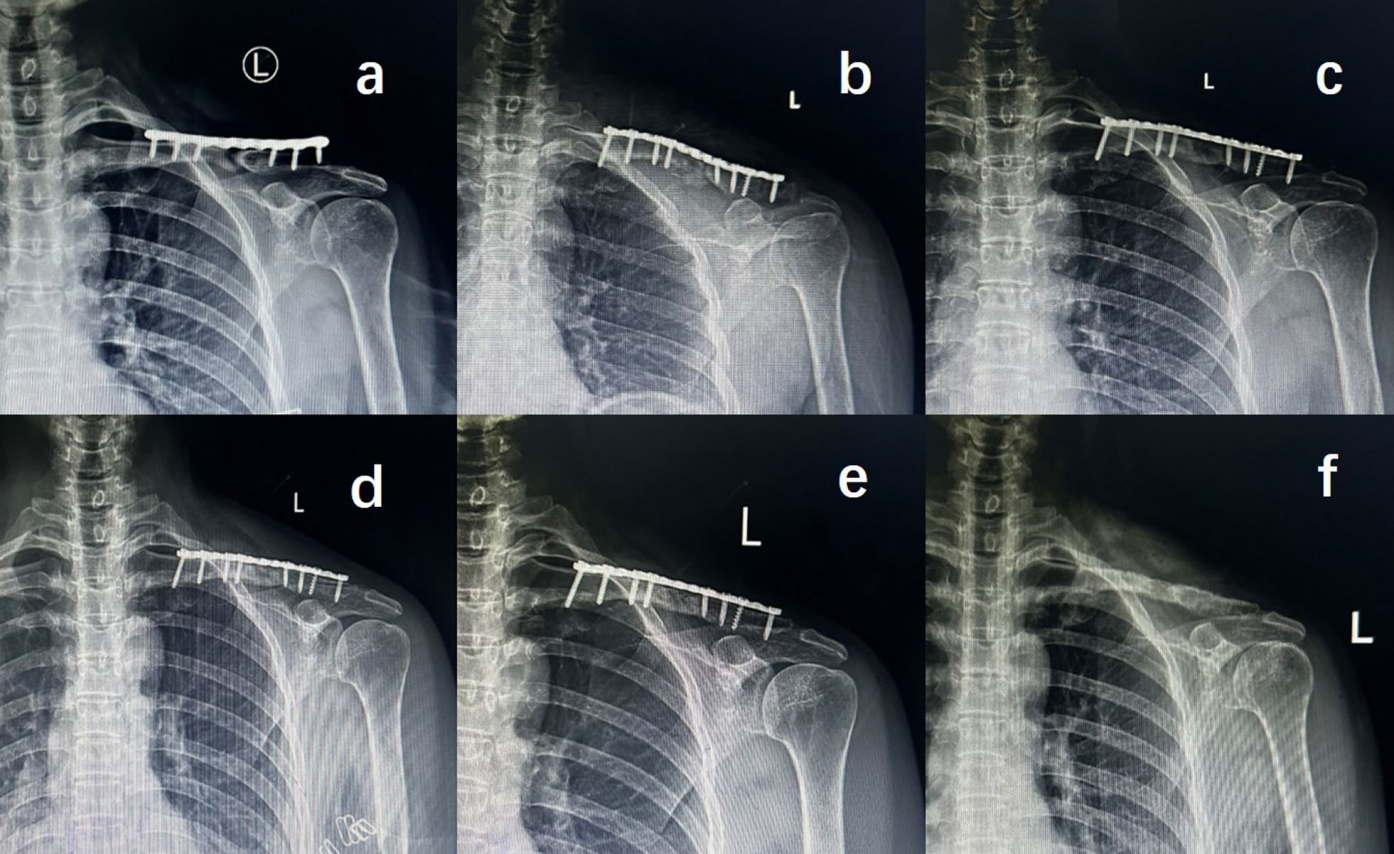
A typical case using the ACGBG technique involving a 54-year-old man with clavicular midshaft nonunion secondary to a fall 2 years prior. **a** Primitive, **b** post-revised, **c** and at 2 months postoperatively. **d** Plain radiographic images showing the union, and continuous sclerotic bone callus passing through the fracture gap, **e** at 15 months post-revision surgery showing stable fracture healing, **f** at day 1 post-plate removal, showing osseous healing at the fracture site. ACGBG, autologous cancellous granular bone grafting

**Fig. 3:**
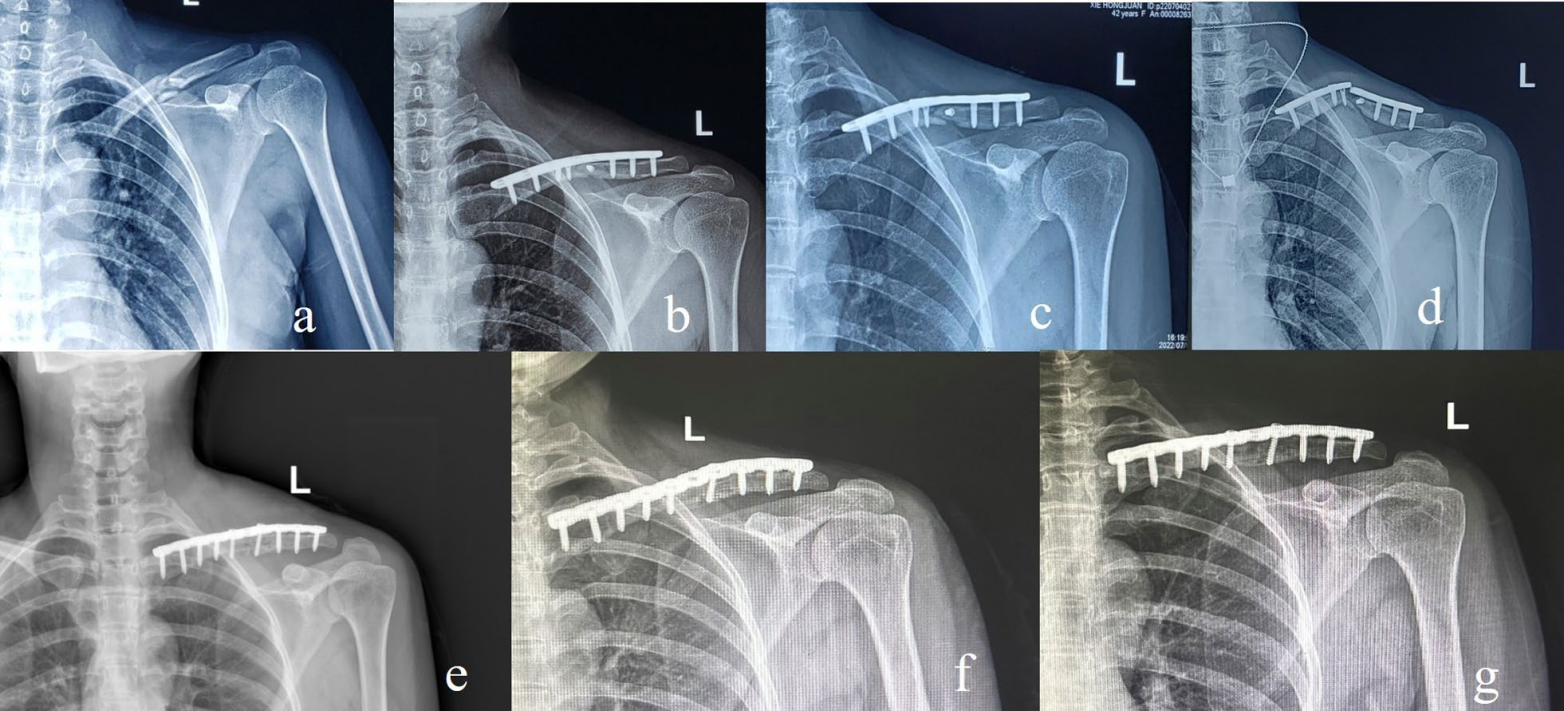
Using the ACSBG technique, a 48-year-old woman sustained a clavicular midshaft fracture as a result of a traffic accident 25 months prior. **a** Primitive, **b** postoperative, **c** and at 5 months postoperatively. **d** Plain radiographic image showing clavicular midshaft bone nonunion and plate breakage, **e** day 1 post-revision surgery, **f** the blurry fracture line, and **g** osseous healing of the fracture site at 3 months post-revision. ACSBG, autologous cancellous structured bone grafting

Bone union was observed at a mean time of 18.6 ± 5.4 (range, 12–32) weeks in the ACGBG group (Fig. 2), which was significantly longer than 15.2 ± 3.0 (range, 12–20) weeks observed in the ACSBG group (*P* = 0.010; Table 2; Fig. 3).

### Complications

In the ACGBG group, one patient’s reconstruction plate broke while carrying a heavy weight 6 months after surgery; this was revised using the ACSBG technique combined with single SP fixation, and the fracture eventually united. One patient experienced persistent pain at the donor site. Two other patients had a wound infection in the donor area and were treated with antibiotic therapy. The incidence of complications was 13.3% (4/30 patients). In the ACGBG group, one patient experienced persistent pain in the donor bone area; one patient had superficial inflammation of the clavicular fracture segment, which occurred on day 3 postoperatively, and recovered following 1 week of antibiotic treatment; and one patient had a stiff shoulder, which improved following 3 months of functional exercise. The complication rate in the ACSGB group was 10.3% (3/29). No statistically significant difference was observed in the incidence of complications between the groups (Table 1).

## Discussion

The middle-third of the clavicle is the most common site in clavicular bone nonunion, located at the junction of the two curves; medially convex anteriorly and laterally convex posteriorly. This site is covered with small muscles and endures strong tension and torsional forces [20]. Therefore, bone nonunion at the clavicular middle site has been challenging for clinical surgeons. Open reduction, SP fixation, and ACGBG provide the best options for treating this type of bone nonunion. In addition, some surgeons have used ACSBG with single SP fixation to treat recalcitrant fracture nonunion, resulting in excellent clinical outcomes [17]. This study focused on the clinical and radiological comparison of two methods of autologous bone grafting with the SP for the treatment of CMAN. Compared with ACGBG, ACSBG combined with single SP fixation resulted in a comparatively shorter time to bone union, better early clinical results, no serious complications during the entire follow-up, and a similar incidence of complications.

### Increasing mechanical stability may be a critical factor for surgical intervention in CAMN

Many implant types have been chosen for the treatment of clavicular fractures, malunion, and nonunion. Intramedullary fixation using Steinmann pins [21], Rush or Hagie pins [22, 23], Kirschner wires or titanium elastic nails [24, 25], extramedullary fixation using external fixators [26], and different plate types have been reported. Direct plate fixation, with or without bone grafting, is currently the most common technique, achieving good clinical outcomes and allowing early rehabilitation [4, 15, 27–30]. Mechanical stability is an absolute requirement for achieving bony union [15]. Some studies have reported surgical techniques used to enhance the stability of the fracture ends when treating atrophic clavicular bone nonunion during surgery. Rollo et al. [15] used an upper anatomical plate combined with an autologous iliac bone graft, and eventually added an allogeneic cortical bone plate on the opposite side of the patient’s bone nonunion sites to augment the stability of the fracture sites. Good radiological results were observed in 43 cases, but 1 case of nonunion, progressed to osseous healing at a mean time of 14 weeks. In 2021, Zhang et al. [29] used a double plate to enhance the mechanical stability of fracture sites, and combined it with autologous bone grafting, to treat 12 patients with atrophic clavicular nonunion. All patients achieved bony healing at an average of 9 weeks postoperatively. These results suggest that enhancing the mechanical stability of the fracture ends is necessary to promote fracture healing and early rehabilitation in patients with atrophic clavicular nonunion. In this study, we opted for single SP fixation combined with bone grafting to treat CMAN for the following reasons: (i) Rollo et al. [15] reported that superior plating achieved greater stability compared with anterior plating; (ii) in a previous study, single SP fixation combined with autogenous structured bone grafting was used as a fixation during recalcitrant CMAN revision surgeries, with excellent clinical outcomes reported [17]; and (iii) the double plate technique involves increased soft tissue resection and bone blood supply destruction, which may influence bone healing.

### Augmenting mechanical stability via implantation of an autogenous structured iliac block in the opened bone slot of the bone nonunion ends

Through opening a slot at the side across the ends of the nonunion, and implanting a complete iliac bone block, the bone was eventually fixed with double-plate fixation, which has been shown to be a good method for treating recalcitrant limb bone nonunion [31]. This result suggests that implanting a complete iliac bone block when opening a bone channel is necessary for the mechanical stability of the fracture ends and bone healing, especially in the treatment of atrophic bone nonunion. However, opening the slot and performing bone grafting when treating CMAN is challenging owing to the smaller clavicle diameter. Our solution was to perform an osteotomy along the medullary cavities at both ends of the clavicular bone nonunion and then move the bone block downward to form an artificial bone groove (Fig. 1). The technique described in this study differs from that previously described by Rollo et al. [15], as follows: (i) the bone graft was placed in an artificial bone slot; therefore, the surface area touching with clavicle was clearly larger than parallel bone end-to-end contact described by Rollo et al.; and (ii) the additional strut graft on the opposite side of the plate used in this study was residual clavicular cortical bone, whereas Rollo et al. used human cadaveric homologous strut grafts. Theoretically, our method should improve bone healing. Therefore, we compared the clinical and radiological results of this novel bone grafting method with those using the conventional bone grafting method.

### Analysis of the results and advantages of implanting a structured bone block into the opened bone slot

Similar bone defect lengths were observed when the operative clearance of the bone nonunion ends in both groups was complete. In this study, the ACSBG technique decreased the fracture healing time by 22.3% (ACGBG group, 18.6 ± 5.4 weeks; ACSBG group, 15.2 ± 3.0 weeks; *P* < 0.05). This could be a key advantage in using the ACSBG technique to treat CMAN. We consider there to be three main reasons for this advantage in implanting a structured bone block into an opened bone slot to promote bone healing in this study. First, implanting a structured bone block between both bone nonunion ends may have increased the mechanical stability compared with granular bone grafting. Second, a structured bone block, used as a bone bridge to connect both healthy segments, provides a path for osteogenic and osteoinductive factors derived from healthy bone tissue (Fig. 1).

The postoperative VAS and DASH scores improved significantly compared with the preoperative VAS scores in both groups. No statistical differences were observed after comparing pre- and 9 month postoperative VAS and DASH scores between the two groups. However, at 3 and 6 months postoperatively, both the VAS and DASH scores in the ACSBG group were significantly improved compared with those in the ACGBG group. Better mechanical stability and earlier bone healing in the ACSBG group may be the two main reasons for these differences at 3 and 6 months postoperatively. Mechanical stability in both groups was consistent when fracture healing occurred 9 months postoperatively, which may explain why both the VAS and DASH scores were similar at that time. In addition, one patient in the ACGBG group had a severe complication postoperatively involving plate breakage at 5 months postoperatively. Bone nonunion in this patient healed after revision surgery using the ACSBG technique with single SP fixation. This suggests that mechanical stability should be prioritized during the surgical treatment of CMAN.

## Study limitations

This study had some limitations. This was a retrospective study, which prohibited the control of variables. The sample size was small during the mid-term period. Our results need to be verified in a larger sample study and with a longer term follow-up period. The ACSBG technique resulted in stronger biomechanical stability than the ACGBG technique, and further biomechanical test results are needed to validate our findings.

## Conclusions

The middle segment of the clavicle has a relatively thin diameter and endures complex biomechanical forces. Therefore, mechanical stability after bone grafting plays a key role in the surgical treatment of CMAN. Compared with the conventional ACGBG technique, the ACSBG with single SP fixation technique resulted in a lower early pain score and a better early functional score and may be a promising treatment option for patients requiring surgery to treat CMAN.

## Data Availability

The data are available from corresponding author if necessary.

## Abbreviations

ACGBG: Autologous cancellous granular bone grafting
ACSBG: Autologous cancellous structured bone grafting
AP: Anteroposterior
CMAN: Clavicular midshaft atrophic nonunion
DASH: Disabilities of the arm, shoulder, and hand
SP: Superior plate
VAS: Visual analog scale
